# Economic crisis, immigrant women and changing availability of intimate partner violence services: a qualitative study of professionals’ perceptions in Spain

**DOI:** 10.1186/s12939-014-0079-1

**Published:** 2014-09-10

**Authors:** Erica Briones-Vozmediano, Andres A Agudelo-Suarez, Isabel Goicolea, Carmen Vives-Cases

**Affiliations:** Department of Community Nursing, Preventive Medicine and Public Health and History of Science, University of Alicante Public Health Research Group, (San Vicente del Raspeig Road), Alicante, (03690) Spain; Faculty of Dentistry, University of Antioquia, (Street 67), Medellín, Colombia; Department of Public Health and Clinical Medicine, Epidemiology and Global Health Unit, and Umeå Center for Gender Studies, Umeå University, Umeå, (SE-901 87) Sweden; CIBER of Epidemiology and Public Health, Carlos III Institute of Health, (Street Melchor Fernández Almagro), Madrid, (28029) Spain

**Keywords:** Intimate partner violence, Immigrant women, Crisis, Qualitative study, Spain, Violencia del Compañero Íntimo, Mujeres inmigrantes, Crisis, Estudio cualitativo, España

## Abstract

**Introduction:**

Since 2008, Spain has been in the throes of an economic crisis. This recession particularly affects the living conditions of vulnerable populations, and has also led to a reversal in social policies and a reduction in resources. In this context, the aim of this study was to explore intimate partner violence (IPV) service providers’ perceptions of the impact of the current economic crisis on these resources in Spain and on their capacity to respond to immigrant women’s needs experiencing IPV.

**Methods:**

A qualitative study was performed based on 43 semi-structured in-depth interviews to social workers, psychologists, intercultural mediators, judges, lawyers, police officers and health professionals from different services dealing with IPV (both, public and NGO’s) and cities in Spain (Barcelona, Madrid, Valencia and Alicante) in 2011. Transcripts were imported into qualitative analysis software (Atlas.ti), and analysed using qualitative content analysis.

**Results:**

We identified four categories related to the perceived impact of the current economic crisis: a) “Immigrant women have it harder now”, b) “IPV and immigration resources are the first in line for cuts”, c) “ Fewer staff means a less effective service” and d) “Equality and IPV policies are no longer a government priority”. A cross-cutting theme emerged from these categories: immigrant women are triply affected; by IPV, by the crisis, and by structural violence.

**Conclusion:**

The professionals interviewed felt that present resources in Spain are insufficient to meet the needs of immigrant women, and that the situation might worsen in the future.

**Electronic supplementary material:**

The online version of this article (doi:10.1186/s12939-014-0079-1) contains supplementary material, which is available to authorized users.

## Resumen

**Introducción:**

Desde 2008, España está atravesando una fuerte recesión económica con consecuencias negativas en las condiciones de vida de los grupos vulnerables de población, las políticas sociales y los recursos. En este contexto, el objetivo de este estudio es explorar la percepción de los profesionales de servicios de atención a la violencia del compañero íntimo (VCI) en España sobre el impacto de la actual crisis económica en estos recursos y su capacidad de respuesta a las necesidades de las mujeres inmigrantes que se encuentren en esta situación.

**Método:**

Se llevó a cabo un estudio cualitativo basado en 43 entrevistas en profundidad semiestructuradas con profesionales de trabajo social, psicología, mediación intercultural, justicia, abogacía, policía y salud trabajando en diferentes servicios de atención a la VCI (tanto públicos como ONG’s) en diferentes ciudades en España (Barcelona, Madrid, Valencia and Alicante) en 2011. Las transcripciones se importaron al software de análisis cualitativo (Atlas.ti) y se realizó un análisis de contenido cualitativo.

**Resultados:**

Se identificaron 4 categorías relacionadas con el impacto percibido de la actual crisis económica: a) “Las mujeres inmigrantes lo tienen ahora más difícil”; b) “Los recursos de VCI e inmigración en el punto de mira de los recortes”, c) “Reducción del personal y aumento de las listas de espera” and d) “Las políticas de igualdad y VCI ya no son una prioridad gubernamental”. Emergió un tema transversal a estas categorías: las mujeres inmigrantes se ven tríplemente afectadas por la crisis, la violencia estructural y la VCI.

**Conclusión:**

Los profesionales entrevistados perciben que la disponibilidad de recursos en España es insuficiente para satisfacer las necesidades de las mujeres inmigrantes, y que esta situación puede empeorar en el futuro.

## Introduction

Violence against women (VAW) is an extreme manifestation of gender inequality in society and a serious violation of fundamental human rights. The United Nations declaration on the Elimination of VAW defines it as any act of gender-based violence that results in, or is likely to result in, physical, sexual or psychological harm or suffering to women, including threats of acts such as coercion or arbitrary deprivation of liberty, whether occurring in public or private life [1]. Intimate Partner Violence (IPV) against women is the most common type of male VAW. It takes place within couples, and the perpetrators are almost exclusively men who are or who have been in an intimate relationship with the woman [2].

IPV against women occurs without exception in all countries, all cultures and at every level of society [3]. In the EU-27, it affects between 20% and 25% of adult women who have ever had an intimate partner [4]. However, within countries, some populations of women may be at greater vulnerable situation such is the case of migrant women due to different factors related to social exclusion: expatriation, lack of legal immigrant status, economic hardship and economic dependence on their partners [5-11].

At the end of 2012, a total of 314,358 persons from abroad set up residence in Spain. Although males traditionally maintained a slightly higher prevalence, women accounted at least for 48% of the total of foreigners that arrived to Spain [12]. The majority of immigrant women living in Spain come from Morocco, East Europe, Ecuador and Colombia. A cross-sectional study of 10,202 women attending primary health care centres showed an IPV of 27.9% in migrants compared to 14.3% in Spanish women [13]. According to the Annual Report of The National Observatory on Violence against women, in 2012, 47% of intimate partner violence-related murders in Spain were produced among migrant women [14].

Several advances in IPV and immigration policies have been achieved in recent years in Spain which have facilitated immigrant women’s access to resources, including the Organic Law/2004 [15]. According to this one, the public sector in Spain (state, regional and local government) is required to provide support to female victims of IPV in the form of information, comprehensive welfare services and free legal aid. This is delivered through 24-hour specialist emergency services, IPV-based courts and specialist units in the state security bodies, and involves professionals from different disciplines (physicians, psychologists, psychiatrists, lawyers, sociologists, educators, social workers, employment specialists). Provided support encompasses health care, emergency services, assistance, accommodation and comprehensive recovery. Support also includes social welfare benefits (unemployment benefit, income support), protection measures, job placement programmes, general and specialised social services (information, counselling, social support, follow up on women’s rights claims, educational support for the family, preventive education and support for vocational training and job seeking), a mobile telecare service, women’s shelters, and priority access to public housing and public residential homes for senior citizens. According to the 2004 law all these resources should be available to all women once an official complaint has been lodged. However, the lack of reports informing on the nationality of women accessing such resources does not allow to point out differences/inequalities on access to such services between immigrant and non-immigrant women exposed to IPV in Spain.

The Organic Law 10/2011 of July 27, 2011 extends the rights of battered immigrant women in an irregular situation, establishing the possibility of requesting permission to reside (including their children) and work due to exceptional circumstances once a restraining order has been granted. Such permission is not definitively approved until criminal proceedings have concluded. They can also apply for provisional authorization to reside and work due to exceptional circumstances at the time of filing the official complaint [16]. NGOs also play a key role in providing care for immigrant women, particularly immigrant associations, since translation and cultural mediation services are not required by law, and are even less likely to be provided now that funding for these resources has been removed. In addition to these State policies, it is important to note that Spain is divided into Autonomous Communities at regional level. Each of the Autonomous Communities has its own government and adopts its own policies, although following some common national policy guidelines. For example, the afore mentioned Organic Law 1/2004 on Comprehensive Protection Measures against Gender-Based Violence is applicable throughout the country, but each region can also have its own law on gender-based violence. Meanwhile, there are 17 Social Services Acts, one for each of the Autonomous Communities. These Acts differ in terms of social rights, social services, and social expenditure. In terms of the health-sector response to IPV whilst all the regions have developed protocols and guidelines, great variation exist between regions in terms of training programs, intersectorial coordination and monitoring systems [17].

The current global recession has prompted a wide range of economic, political and social reforms in Europe and Spain which involve cutbacks in social investment [18-20]. These policies reflect the priority given by governments to reduce their fiscal deficits by cutting back on social expenditure [21]. The concomitant dismantling of the welfare state means that social and health care services are the first to be affected by government austerity measures [22,23]. For example, between 2006 and 2011 the percentage of the total Spanish State Budget allocated to health care fell from 35.2% to 33.7% (almost 2 points), representing a reduction of more than 8 million Euros [19]. Other institutional reports have indicated that per capita social expenditure in the period 2009–2011 fell significantly by a state-wide average of 4.4% [24]. In 2011, the at-risk-of-poverty rate stood at 21.8% for the population of Spain, and one in four people under the age of 16 were living below the poverty line [25].

Austerity measures are harming the social well-being of the populations affected [26,27]. Previous research has shown that due to their effects on the labour market and resource access, economic downturns have negative consequences for public health [23,28,29]. Similarly, social inequalities between population groups widen during recessions; rising unemployment and poverty hit the most disadvantaged hardest and increase their vulnerability [30,31]. In the particular case of the immigrant population in Spain, it has already been observed that those who still do not return to their country of origin are among the many unemployed who struggle to meet very basic needs for food, housing, health, education, and social protection [32].

Implementation of public IPV policies depends heavily on the service providers [33], and they can also be an important source of information about the impact of the economic crisis on social strategies targeting immigrant women. Therefore, an analysis which takes into account the perspectives of those involved might identify the factors which could prevent the crisis from affecting public health [23,34]. The effects of the reforms prompted by the current economic crisis on IPV resources have not been explored in Spain [35]. An exploration of service providers’ opinions and concerns about areas under threat could serve as a first step in formulating policy and research actions [36-38]. Meanwhile, at an organisational level, it is necessary to study how professionals tackle changing conditions [39].

This study formed part of a wider research project examining the determinants of access to the health and social services available in Spain for tackling IPV in general, and among immigrant women in particular [40]. Concern about anticipated spending cuts prompted by the crisis and their impact on resources was a theme that emerged from the interviews, since the fieldwork was conducted in a context of an economic recession (March-December, 2011). Therefore, the aim of the present study was to explore IPV service providers’ perceptions concerning the impact of the economic crisis on both the changing availability of IPV-related services and their own capacity to respond to immigrant women experiencing IPV in Spain.

## Methods

### Design and participants

Twenty nine in-depth personal interviews were conducted and four multiple interviews (interviews with two or three interviewees) held with a total of 43 professionals working in NGOs, public institutions and specialised services who gave legal, social, health, employment, family and psychological support to immigrant women (Additional file 1: Table S1). The interviewees came from different cities in Spain (Barcelona, Madrid, Valencia and Alicante). Those cities concentrate 46% of the migrant women population of Spain [12]. We aimed to gain an integrated overview of service provision by including multiple voices and asking different kinds of questions to different participants, adjusting each interview to address the questions appropriate to that participant’s profile [41].

Theoretical sampling was carried out, including participants with different profiles working in public and private institutions and NGOs capable of contributing to our research question. The first strategy employed to recruit participants was to contact a shelter in Alicante. Next, we recruited professionals working with NGOs via a social worker, and also employed the snowball method - in each interview, information was requested concerning possible contacts.

### Data collection

The interview guide included a series of topics to be discussed during the interview. A list of lines of inquiry was drawn up to provide guidelines for interaction with interviewees and subsequent organisation of the information. There was no predetermined sequential order, and questions were open-ended. The interview guide was drawn up after reviewing the literature, and also reflected the experience and knowledge of the research team (See Interview guide on Additional file 2).

The interview was divided into two sections, with an opening question concerning the professional competences of the interviewees’ specialist areas in the provision of support to immigrant battered women, and a closing question about their general evaluation and the possibility of improving support to immigrant battered women. The first section dealt with their experience with immigrant battered women and the problems encountered, and the second with the interventions carried out and their perceptions about immigrant women’s satisfaction with these interventions and resources.

The interviews were carried out by one member of the research team and lasted between 35 and 90 min. All the interviews were conducted in Spanish, which was the mother tongue for both interviewer and participants. The professionals were interviewed at their workplace. We continued to conduct interviews until data saturation was achieved, meaning that no new information related to the research question was emerging [42]. In keeping with the principles of the Declaration of Helsinki and the Belmont Report, the purpose and procedure of the study were explained, an opportunity to ask questions was provided, and written informed consent was obtained from each participant prior to data collection. During the multiple interviews, the interviewer stressed the importance of respecting others’ opinions and maintaining the privacy of what was said within the group. Ethical approval was sought from the Ethics Committee of the University of Alicante.

### Data analysis

We digitally recorded the interviews and then transcribed them verbatim. Each of the authors examined all the interviews independently, and then met to compare and combine their analyses. Transcripts were imported into qualitative analysis software (Atlas.ti) as a tool for organizing the information, through which the authors analysed the transcripts using qualitative content analysis [43]. Through the texts, we identified meaning units of a sentence or paragraph with the same content. Then we drew up condensed meaning units, namely summarised versions of the initially identified units. These condensed versions were used to generate codes, which were grouped together in order to create categories reflecting the manifest content of the text, namely what the providers explicitly expressed about the impact of the crisis on IPV resources in Spain. Finally, a cross-cutting theme emerged from the categories that reflected the latent content of the interviews.

## Results

The analysis of the interviews with service providers revealed four categories related to the negative impact of the reforms prompted by the economic crisis, as perceived by professionals: a) “Immigrant women have it harder now”, b) “IPV and immigration resources are the first in line for cuts”, c) “Fewer staff means a less effective service” and d) “Equality and IPV policies are no longer a government priority”. These categories described the perceived and anticipated effects of changes in the allocation of resources for IPV support (Additional file 3: Figure S1).

A cross-cutting theme emerged from the categories, which expressed the latent content of the interviews: *Immigrant women are triply affected; by IPV, by the economic crisis and by structural violence.* Service providers perceived immigrant women to be particularly vulnerable to IPV, on the one hand, and to the effects of the recession on the other. In the opinion of the professionals interviewed, the economic crisis could lead to structural violence against immigrant women who experience IPV; they anticipated a reduction in support resources for these women as a result of budget cuts, and consequently, fewer opportunities for the empowerment which would enable them escape from situations of IPV (Additional file 3: Figure S1).

### “Immigrant women have it harder now”

Service providers perceived additional obstacles for immigrant women attempting to leave violent relationships, which stemmed from the migration process: no legally valid documentation, language barriers and the lack of support networks, among others. These factors may provoke a situation of greater vulnerability compared with Spanish women and can act as barriers to resource access, making it more difficult for immigrant women to break free from situations of IPV. Furthermore, any difficulties arising from the migration process are exacerbated by the economic crisis.

The recession has hit immigrants much harder than Spanish people because in most cases their situation is much more complicated: they’re more vulnerable, they have fewer social networks, I mean, everything is more complex in these cases. (Interview 18 - psychologist)

The professionals interviewed considered that being an immigrant frequently entailed exploitative working conditions and socio-economic insecurity. In addition, the economic crisis has intensified women’s economic insecurity.

Another consequence of being an immigrant is lack of money, and it’s much worse now with the economic crisis because there’s a lot of non-payment, [they] don’t have any income, they’ve got children, they’re living in very bad conditions, all packed into one rented room… (Interview 33 - judge)

As long as the employment situation stays the same, you know that any immigrant, or any woman, with no training, support, personal resources, family or social services, is going to be exposed to all kinds of exploitation. (Interview 31 - social workers and psychologists)

They believe that the recession in Spain is having a negative effect on immigrant women’s opportunities for economic empowerment, because high unemployment rates and scarce job opportunities have reduced immigrant women’s possibilities of entering the workforce and achieving the economic independence. Thus, not only are their opportunities for attaining independence diminished, but their ties of economic dependence on the abuser are strengthened, rendering it more difficult for them to leave a violent relationship.

And the labour market, who is going to be hit hardest in the labour market? Women, and even more so, immigrant women, make no mistake. I mean, the groups in the most vulnerable situations are the ones who get hit the hardest. And in the labour market, women occupy a more tenuous position, and the position of immigrant women is even more precarious. So obviously it’s indirectly going to affect women’s recovery process. (Interview 25 – social worker)

### “IPV and immigration resources are the first in line for cuts”

At the time of the interviews, the service providers were already reporting shortages and a lack of the resources necessary to provide support in cases of IPV, even before the reforms prompted by the economic crisis had begun to take their toll.

Both you and I know perfectly well that services in the public sector do not work, and especially nowadays. (Interview 10 - mediator)

Health services, for example, the way they’re set up they’re no use. They’re no use because of the timetables, are they any use to you? (Interview 18 - psychologist)

They expressed concern about resource provision as the recession deepens, anticipating a reduction in IPV services and resources due to budget cuts as a result of dwindling funding for these purposes.

There aren’t many resources, and now that the welfare state is being dismantled there’ll be even fewer, we’re being merged, I don’t know how long these specialist services will continue to exist, and the future looks bleak in that respect. (Interview 29 - social worker)

They felt that cutbacks in social programmes would affect the availability of essential resources for immigrant women in situations of abuse. There was also concern that economic and social cuts by the new government would adversely affect the allocation of IPV resources nationally, as would the government’s policies on changing the administrative structure of assistance.

Let’s see, I think the economic situation means that our response capacity is lower because there are fewer resources, and there will definitely be fewer resources in the future. (Interview 31-social workers and psychologists)

If we’re noticing it now in associations, just imagine what’s going to happen in terms of grants, just imagine. (Interview 25 – social worker)

Providers also suspected that there would be cutbacks in financial aid such as income support for battered women, an essential resource to ensure women’s economic independence from aggressors. However, there were also more positive opinions expressed about the continuity of IPV support despite the crisis, linked to the personal motivation of some professionals rather than to faith in the structures. In spite of the cuts they anticipated as a result of the economic crisis, some participants expressed positive feelings, mainly about their work as professionals supporting immigrant women. As one of the participants stated “Large or small, every stone helps build a wall” (Interview 5- social worker).

I think that many things are being scrutinised, although it’s also true that the political situation at the moment is difficult. A lot’s been changed, there are plans to improve, I think that even from within the service there are new proposals, but of course with the budget as it is, on hold… a lot’s been done and must continue to be done, but it’s a difficult time, politically and economically, and I really don’t know how it will all end. (Interview 32 - psychologist)

Participants expected or anticipated that the scope of activities would continue as before, but that any new programmes or initiatives would be cancelled or delayed due to the crisis. Despite these difficulties, the professionals attempted to compensate for cuts with their own personal efforts and motivation, and continued working.

Obviously there are strengths and good points, a lot’s been done in all this time and progress has been made even though they’re cutting back on resources and we can’t do the practical things that we were doing before, but there have been huge advances in education. (Interview 31 - social workers and psychologists)

### “Fewer staff means a less effective service”

Professionals criticised the lack of human resources in IPV support services and highlighted the need for more specialist staff to meet the specific needs of immigrant women, such as translators or cultural mediators.

We don’t have mediators, we don’t have interpreters. If someone goes to the doctor and you don’t understand Arabic, and their child is in school, obviously you aren’t going to talk in Arabic because you don’t speak it, so how do you communicate with this person? What’s more, what do you know? What do you know about the culture this person comes from? (Interview 18 - psychologist)

Staff cuts as a result of reduced budgets impaired the quality of the services offered. Interviewees thought that cuts in IPV services would have an impact on the quality of the support given to the immigrant battered women, because fewer staff meant more work for the remaining professionals and less time for each individual client.

For example, there’s this long wait between one appointment and another, you need a lot of resources for the woman to retain the trust that you’ve tried to instil in her, you know? So that she lets you help her. You need a lot of resources. I don’t care about the crisis, you need one social worker per case, not fourteen cases per social worker because that gets you nowhere, but hey, the reality’s different. (Interview 10 - mediator)

Another result of the recession is that working conditions in IPV services have become harsher, with increased workloads, a freeze on recruitment and reduced shifts and salaries. These shortcomings were highlighted as an obstacle to interventions with women, hampering the provision of effective attention.

with the hours we do and the low salary, I don’t think our work is really appreciated, given all the responsibility we have and all the work we have to do. The service is put out to tender, and so they award it to the company that presents the cheapest project and so on, or they don’t get paid on time, sometimes months and years go by without paying the company, and that’s detrimental to the people working there. (Interview 29 - social worker)

### “Equality and IPV policies are no longer a government priority”

The professionals interviewed were concerned and uncertain about the immediate future in Spain as regards IPV policies, anticipating reversals in policy prompted by the economic crisis and the arrival of a new government different to the one that enacted the IPV Law.

Well, they say they want to change the law on gender-based violence in the courts and I don’t know in what way they want to do that, the new government, I’ve got no idea. So I suppose we’ll have to wait and see. (Interview 33 - judge)

They expressed concern about possible amendments to IPV legislation and the potential effects on social policies aimed at the immigrant population of the new government’s reforms in the context of an economic crisis. They felt that this situation represented a step backwards in terms of the advances that Spanish society had achieved through the time and efforts of social and individual stakeholders, such as the people working in IPV services themselves. For example, the politicians were threatening to change the specialised IPV courts or the law on IPV, which the politicians were threatening to change.

Specialised IPV courts have been a great step forwards, I’m scared of what’s going to happen, I’m not at all clear what they’re going to do. I don’t know, what with the crisis, the cuts, I don’t know what they’re going to do… (Interview 31 - social workers and psychologists)

Participants warned that economic crises threaten advances in social policies and equality. They believed that equality policies constituted a structural battle against gender-based violence, and feared that these would stagnate or even be reversed, largely to the detriment of victims. They felt that gender equality, the fight against IPV and the specific needs of the immigrant population were not priorities for policy-makers.

What’s more, when there’s an economic crisis, the government pushes equality to the sidelines, and we’re always hearing that equality is a cross-cutting objective that should be in place everywhere, in culture, in the economy, in politics, in everything, but it’s no longer a priority and we’re already seeing that. (Interview 31 - social workers and psychologists)

## Discussion

This study points out two important aspects regarding how professionals perceived the effect of austerity measures on the resources available for immigrant women exposed to IPV. First, they considered that the economic crisis was hitting harder immigrant women compared to non immigrant women. They also considered that the crisis had led to cutbacks of funding that were affecting the quality of the services they were offering to women exposed to IPV in general, and to immigrant women in particular. Secondly, they were pessimistic in terms of the future. Policy changes and economic cuts in health and social services were identified as the main threats to IPV services. These changes were expected to set drawbacks in policies and programs related to migrants’ rights and gender equality.

The results are consistent with those reported in other studies, in that they indicate that the economic crisis may contribute to increasing the risk of social exclusion among certain populations, which in the specific case of this study would imply increased vulnerability among immigrant women to remaining in a situation of IPV [44]. It is well known that the context of migration poses additional difficulties associated with situations of greater social vulnerability, such as the lack of a residence permit, existence of language barriers and the lack of support networks in the event of difficulties or problems [45,46,10]. The crisis adds to this burden by increasing unemployment rates among immigrant women, with the consequent deterioration in their standard of living, increased economic dependence on violent partners and decreased chances of finding employment.

The results indicate that service providers experience an overwhelming amount of work followed by burnout, which in turn affects the care given to women. On the one hand, the professionals interviewed were already beginning to experience funding cuts, in line with the general tendency among governments in times of recession to reduce public spending by implementing changes in funding, prioritisation and governmental regulation, and by decentralising health services [38]. That could be explained because they are considered as “unproductive” components of government spending [20]. In consequence, and as the results show, such services are subject to staff cuts, leading to an overload of work and the risk of burnout among the staff who are still employed. Other studies have indicated that one of the main barriers to the provision of effective care in cases of IPV is understaffing, given that in times of economic recession it is more difficult to ensure the quality of care by hiring interpreters or staff with specialised training in matters related to immigration and intercultural skills [47,48]. This reduction in staffing levels is worrying because when other resources are being cut at national level (macro level) or regional level (meso level), the professional role of human resources (micro level) becomes decisive [47,49].

As regards future expectations, the service providers interviewed in this study anticipated that IPV services and services aimed at vulnerable populations were particularly at risk in the current economic climate. Participants thought that the new government would cut public services, and that the lack of economic resources would lead to a reduction in infrastructures and staff and the closure of some services. This would have a negative impact on their ability to respond to IPV, especially in the case of immigrant women because these are exposed to multiple factors of social exclusion. These predictions have subsequently been confirmed: several services frequently utilised by immigrants, such as NGOs, are closing due to budget cuts, and immigrants’ free access to health services has been curtailed [27,50]. Current health reforms will hinder the protection of immigrant women living in Spain –those without official immigrant status and experiencing IPV- as they will be excluded from the health system. Consequently, they will not be able to provide crucial evidence when they need to apply for a restraining order against their aggressor. Both their perceptions and the confirmed facts indicate that the effect of macroeconomic and neo-liberal political reforms is to impose structural violence on vulnerable populations because, on the one hand, such reforms increase individual and community vulnerability to specific problems, and on the other hand, they lead to a dismantling of already debilitated public services [51].

As well, they were right about the change of government and the impact on IPV support policies and strategies. In Spain, from 2004 to 2011, the previous socialist government was heavily committed to the implementation of IPV services, especially following enactment of Organic Law 1/2004 on gender-based violence. These facts confirm that, what the professionals considered to be consequences of the crisis, however, were in fact the effects of government priorities prompted by the recession, priorities which have meant that today, the health and social services are facing financial constraints [36]. Proof of this is that spending on equality policies has been changed and some of these policies are being reformulated to the detriment of women’s fundamental rights, such as the right to abortion. In this sense, there is a need for further research about how policies towards mitigating IPV in immigrant women operate in other European countries and whether EU countries have introduced similar cuts.

## Conclusions

These findings lead us to conclude that the policy changes prompted by the crisis hinder effective coverage of services for women victims of IPV in general, and more specifically, for immigrant women in this situation. The present study revealed that service providers tended to think that IPV services and programmes, and especially those for immigrant women, were under threat, since politicians might considered them the most dispensable aspect of the health and social services. Although the maintenance of services and programmes for immigrant battered women is a question of equality, the interviewees felt that this principle was being flouted as a result of government responses to the economic crisis.

### Limitations and strengths

This study has several limitations. First, the issues addressed in this paper emerged spontaneously during the interviews with service providers; the impact of the recession being not the main focus of the study. During data collection and interview analysis alike, the issue of the possible effect on IPV resources of changes prompted by the crisis emerged as a strong theme; we therefore decided to explore this issue further in the present study. Second, this study focused on professionals’ perceptions about what might happen, because the interviews were conducted before the change of government and implementation of the first spending cuts. As the participants had anticipated, several reforms were implemented which affected health and social services. Nevertheless, the reforms had begun some time previously; for example, the responsibilities of the Spanish Ministry for Equality were reduced and the Ministry itself was absorbed by the Spanish Ministry for Health and Social Policy in 2010, and several regional Institutes for Women began to disappear. Furthermore, key national NGOs working in IPV also disappeared as a consequence of the lack of public funding [50].

To the best of our knowledge, this study represents the first attempt to obtain and synthesise service providers’ opinions about the impact of the current economic crisis on the IPV support services in which they work. The methodology employed in this study and the results on IPV resources are not statistically generalizable; however, they can be used to theorise about how professionals perceive a deterioration in services in times of economic crisis [52]. Thus, the study could be used to inform policy-making in Spain, especially in the current context of severe recession, and may also be of interest to other European countries facing similar challenges in resource allocation [47].

## Additional files

Additional file 1: Table S1. Professionals interviewed (n=43). Additional file 2
**Outline of questions to ask during the interviews*.**
Additional file 3: Figure S1. Categories and theme identified in the interviews with participants (n=43).
