# Supplementary material for: Economic crisis, immigrant women and changing availability of intimate partner violence services: a qualitative study of professionals’ perceptions in Spain
Source: Int J Equity Health. 2014 Sep 10;13:79. doi: 10.1186/s12939-014-0079-1 (PMC4172960; doi:10.1186/s12939-014-0079-1)
Supplement: Additional file 1: Table S1. — Professionals interviewed (n=43). [file 12939_2014_79_MOESM1_ESM.doc]

| **Table 1.**  Professionals interviewed (n=43) | | | |  |
| --- | --- | --- | --- | --- |
| **Place of work** | **Interview code** | **Sex** | **Professional Profile** | **City/ Region** |
| **Social Services** | 1 | Female | Social Educator | Alicante/Valencian |
|  | 2 | Female | Psychologist | Alicante/Valencian |
|  | 3 | Female | Psychologist | Alicante/Valencian |
|  | 4 | Female | Social Educator | Alicante/Valencian |
|  | 5 | Female | Social Worker | Alicante/Valencian |
|  | 6 | Male | Social Educator | Alicante/Valencian |
|  | 11 | Female | Lawyer | Alicante/Valencian |
|  | 12 | Female | Social Worker | Alicante/Valencian |
|  | 24 | Female | Psychologist and Social Worker | Barcelona /Catalonian |
|  | 25 | Females | 1 Social Worker and 1 Anthropologist | Barcelona /Catalonian |
|  | 28 | Female | Social Worker | Madrid/ Madrid |
|  | 29 | Female | Social Worker | Madrid/ Madrid |
|  | 30 | Female | Psychologist | Madrid/ Madrid |
|  | 32 | Female | Psychologist | Barcelona /Catalonian |
| **Associations/NGOs** | 7 | Females | 1 Social Worker and 1 Sociologist | Alicante/Valencian |
|  | 8 | Female | Social Worker | Alicante/Valencian |
|  | 9 | Female | Lawyer | Alicante/Valencian |
|  | 10 | Female | Mediator | Alicante/Valencian |
|  | 13 | Females | 4 former IPV victims | Alicante/Valencian |
|  | 14 | Female | Lawyer | Alicante/Valencian |
|  | 15 | Female | Mediator | Alicante/Valencian |
|  | 16 | Female | Health worker | Alicante/Valencian |
|  | 17 | Female | Lawyer | Alicante/Valencian |
|  | 18 | Female | Psychologist | Alicante/Valencian |
|  | 19 | Female | Social Worker | Alicante/Valencian |
| **Law/Police** | 20 | Female | Lawyer | Alicante/Valencian |
|  | 21 | Female | Police Officer | Alicante/Valencian |
|  | 22 | Male | Police Officer | Alicante/Valencian |
|  | 23 | Male | Lawyer | Alicante/Valencian |
|  | 26 | Female | Police Officer | Alicante/Valencian |
|  | 27 | Female | Lawyer | Alicante/Valencian |
|  | 31 | Females | 2 Psychologists and 3 Social Workers | Madrid/ Madrid |
|  | 33 | Female | Judge | Barcelona /Catalonian |
