# Supplementary material for: Economic crisis, immigrant women and changing availability of intimate partner violence services: a qualitative study of professionals’ perceptions in Spain
Source: Int J Equity Health. 2014 Sep 10;13:79. doi: 10.1186/s12939-014-0079-1 (PMC4172960; doi:10.1186/s12939-014-0079-1)
Supplement: Additional file 2 — Outline of questions to ask during the interviews*. [file 12939_2014_79_MOESM2_ESM.doc]

| **Appendix 1. Outline of questions to ask during the interviews*** |
| --- |
| SECTION I. OPENING QUESTIONS |
| What professional experience do you have of working with immigrant populations in general, and with immigrant women in particular? In what contexts have you gained this experience? |
| SECTION II. ACCESS TO RESOURCES |
| In your work with immigrant women, have you seen any cases of abuse? Or do you know if a co-worker has come across such a case? |
| Based on your professional experience, what triggers a woman to disclose that she is experiencing abuse? |
| What kind of support does a woman who says she is experiencing abuse receive? *(employment, education, social, etc.)* |
| And what about women who do not disclose it? Do you remember any case in which you suspected abuse was occurring even though the woman did not say so? |
| What is the protocol in these situations? |
| In your professional work, what nationalities of women have you worked with? |
| Have you noticed any differences in the process with women immigrants of different nationalities? |
| If we compare immigrant women experiencing abuse and requesting support, and immigrant women who are not experiencing abuse, what differences stand out for you? And what similarities? |
| SECTION III. CASE FOLLOW-UP |
| *Now let's talk about what women say they did before coming here...*What steps did they take? What response did they receive? |
| What do you know about what happens to these women once they have contacted some kind of service? How would you assess these previous experiences of seeking help elsewhere or from other professionals? How do you know this? |
| *Returning to the aggressor*: |
| Have you had any experience of women returning to their aggressor? |
| In your opinion, what drives them back to their aggressor? |
| On the other hand, what can protect them, i.e. what could help them sustain their decision to leave a violent relationship? |
| *Filing a complaint*: |
| At what point in your work with these women do they file a complaint about abuse? Have you ever had any such experience? What makes them take this step? |
| Based on your experience, what aspects could be considered the most frequently mentioned by these women when filing a complaint? |
| Have you had any experience of women withdrawing their complaint? Could you tell me about this aspect? |
| SECTION IV. PERCEPTIONS OF WOMEN IMMIGRANTS |
| *Let's talk about how these women see and interpret their experience of abuse...*What problems do they mention? How do they deal with them? |
| What response do they obtain when seeking help? What do they think about the available resources? |
| *Now let's talk about the administrative situation*. Do you think that this has an influence when dealing with abuse? Could you tell me about this? Do you remember any case in particular? |
| Based on your experience and the women's statements, what other aspects might influence immigrant women's experience of abuse? |
| SECTION V. SUGGESTIONS FOR IMPROVEMENT |
| In your opinion, how could work with immigrant women experiencing abuse be improved? |
| Do you think that there is any cultural characteristic that professionals working in services related to gender-based violence among Moroccan or Latin American women should be aware of? |
| Could you tell me the best experience you've had of helping a woman in this kind of situation? |
| Would you like to add anything else that you feel is important to include in this interview that we haven't discussed? |
| *Ask about possible contacts for future interviews* |

- *These questions were not always asked literally or in the same order, but provided an indication of issues to address, and were adapted to each individual case.
