# Supplementary material for: Economic crisis, immigrant women and changing availability of intimate partner violence services: a qualitative study of professionals’ perceptions in Spain
Source: Int J Equity Health. 2014 Sep 10;13:79. doi: 10.1186/s12939-014-0079-1 (PMC4172960; doi:10.1186/s12939-014-0079-1)
Supplement: Additional file 3: Figure S1. — Categories and theme identified in the interviews with participants (n=43). [file 12939_2014_79_MOESM3_ESM.doc]

| Theme | Immigrant women are triply affected; by IPV, by the crisis, and by structural violence. | | | |
| --- | --- | --- | --- | --- |
| Categories | Immigrant women have it harder now | IPV and immigration resources are the first in line for cuts | Fewer staff means a less effective service | Equality and IPV policies are no longer a government priority |
| Selected codes | Greater vulnerability  Irregular migration situation  Socio-economic insecurity  Barriers to resource access | Budget cuts  Concern about shortages  Anticipated reduction on services  Administrative changes | Reduction of staff  Slower service  Lack of specialist staff  Increased workloads | Reversals in policy  Concern about policy changes  Step backwards  Equality policies threatened |

Figure 1. Categories and theme identified in the interviews with participants (n=43)
